# Very Dangerous

**Published:** 1900-06

**Authors:** 


### Very Dangerous.

Singleton:—"Do you agree with the doctors who consider kissing dangerous?"

Benedict:—"Oh, yes."

Singleton:—"What dread effect do you think is likely to arise from it?"

Benedict:—"Marriage."

